# Black phosphorus-based photothermal therapy with aCD47-mediated immune checkpoint blockade for enhanced cancer immunotherapy

**DOI:** 10.1038/s41377-020-00388-3

**Published:** 2020-09-15

**Authors:** Zhongjian Xie, Minhua Peng, Ruitao Lu, Xiangying Meng, Weiyuan Liang, Zhongjun Li, Meng Qiu, Bin Zhang, Guohui Nie, Ni Xie, Han Zhang, Paras N. Prasad

**Affiliations:** 1 Shenzhen International Institute for Biomedical Research, Shenzhen, 518116 Guangdong PR China; 2 Shenzhen Institutes of Advanced Technology, Chinese Academy of Sciences, Shenzhen, 518055 Guangdong PR China; 3 School of Basic Medical Sciences, Guangzhou Medical University, Guangzhou, 511436 Guangdong PR China; 4 Key Laboratory of Optoelectronic Devices and Systems of Ministry of Education and Guangdong Province, Institute of Microscale Optoelectronics, and Otolaryngology Department and Biobank of the First Affiliated Hospital, Shenzhen Second People’s Hospital, Health Science Center, Shenzhen University, Shenzhen, 518060 PR China; 5 Key Laboratory of Marine Chemistry Theory and Technology (Ocean University of China), Ministry of Education, Qingdao, 266100 PR China; 6 Department of Chemistry, Institute for Lasers, Photonics, and Biophotonics, University at Buffalo, StateUniversity of New York, Buffalo, NY USA

**Keywords:** Biophotonics, Biomaterials - vaccines

## Abstract

Here, we describe a combination strategy of black phosphorus (BP)-based photothermal therapy together with anti-CD47 antibody (aCD47)-based immunotherapy to synergistically enhance cancer treatment. Tumour resistance to immune checkpoint blockades in most cancers due to immune escape from host surveillance, along with the initiation of metastasis through immunosuppressive cells in the tumour microenvironment, remains a significant challenge for cancer immunotherapy. aCD47, an agent for CD47/SIRPα axis blockade, induces modest phagocytic activity and a low response rate for monotherapy, resulting in failures in clinical trials. We showed that BP-mediated ablation of tumours through photothermal effects could serve as an effective strategy for specific immunological stimulation, improving the inherently poor immunogenicity of tumours, which is particularly useful for enhancing cancer immunotherapy. BP in combination with aCD47 blockade activates both innate and adaptive immunities and promotes local and systemic anticancer immune responses, thus offering a synergistically enhanced effect in suppression of tumour progression and in inducing abscopal effects for inhibition of metastatic cancers. Our combination strategy provides a promising platform in which photothermal agents could help to enhance the therapeutic efficacy of immunotherapy.

## Immunotherapy: enhancing the therapeutic effectiveness of photothermal cancer treatments

By combining photothermal therapy and immunotherapy, scientists have improved cancer treatments. In cancer immunotherapy, the body’s immune system is artificially boosted to fight cancer. Some cancers, however, can protect themselves from immunotherapies by stimulating immune checkpoint targets, which regulate the immune system and prevent it from attacking non-cancerous cells. Checkpoint blockade immunotherapy helps the immune system to recognise and attack cancer cells. However, it is ineffective in treating patients suffering from most solid tumour cancers like melanomas. Now, a team of scientists, led by Paras Prasad from the State University of New York, have used a combination of black phosphorus-based photothermal therapy and antibody immunotherapy to enhance the therapeutic efficiency of the treatment. By exerting a synergistically enhanced antitumor effect, the technique has proved effective in suppressing the proliferation and growth of cancer cells.

## Introduction

Cancer immunotherapy, also called immuno-oncology, is a treatment strategy that artificially boosts the body’s own immunity to battle cancer. Immune checkpoint blockade is the most promising therapy for the activation of antitumor immunity that targets the immune checkpoint, which is the key regulator overexpressed in cancer cells to escape the body’s immune system^1^. Currently, FDA-approved immune checkpoint blockades target the molecules PD-1^2,3^, PD-L1^4,5^ and CTLA4^6,7^, which play pivotal roles in the regulation of T-cell activities, for the treatment of solid, metastatic and haematological tumours. However, the therapeutic efficiency of immune checkpoint blockades remains relatively low for most cancer patients, especially those suffering from solid tumours.

A lack of recognisable antigens on the tumour cell surface, defects in tumour-specific antigen presentation, the absence of infiltration of cytotoxic T lymphocytes (CTL), severe T-cell exhaustion and immune escape from host surveillance contribute to the initial resistance to immune checkpoint blockades. The immunosuppression status within most tumours is a major challenge because no efficient and durable antitumour immune response can be established or maintained. Even worse, these immunosuppressive cells can promote tumour immune escape, progression and metastasis. Extensive evidence has demonstrated that the major immunosuppressive cells promoting tumour progression comprise myeloid-derived suppressor cells, tumour-associated macrophages (TAMs), regulatory T cells and T helper 17 cells^8,9^. TAMs, as one of the most abundant immune cells, are dependent on the factors present in the tumour microenvironment^10^ and characterised by their protumoural functions, such as tumour growth promotion, progression of invasion and metastasis and even induction of angiogenesis^11^. TAMs can secrete a series of chemokines and cytokines that directly or indirectly suppress the activity of CD8^+^ and CD4^+^ T cells to facilitate tumour immune escape^12^. Macrophages infiltrating the tumour microenvironment are composed of two subtypes: M1-like and M2-like macrophages. The former are classically activated macrophages that exert antitumour functions, such as antigen presentation and lysis of cancer cells. The latter are alternatively activated macrophages, exhibiting protumoural effects by directly participating in cancer cell proliferation, invasiveness, and progression along with immunosuppression^13–15^. The repolarization of TAMs from M2-like to M1-like macrophages has been shown to be a promising target for controlling tumour progression^16^.

Macrophages as effector cells of cancer immunotherapy may have therapeutic potential because of their potent phagocytosis ability. CD47, a self-recognition protein, is ubiquitously expressed on human cells and upregulated in many different tumour cells^17^. By interacting with signal regulatory protein-alpha (SIRPα)^18^, which is expressed on macrophages, CD47 can realise the function of ‘don’t eat me’. Binding of CD47 of tumour binding to SIRPα inhibits the phagocytosis of macrophages to evade innate immunological eradication as well as subsequent adaptive immunity^19,20^. CD47/SIRPα axis blockade agents such as anti-CD47 antibodies (aCD47) have demonstrated excellent efficacy against lymphocytic leukaemia^17^, hepato cellular carcinoma^21^, acute myeloid leukaemia^19^, aggressive metastatic leiomyosarcoma^22^ and solid tumours^23^. Currently, a CD47 antagonist (Hu5F9-G4) has completed its phase 1 trial for treating patients with haematological malignancies (http://clinicaltrials.gov identifier: NCT01410981) and solid tumours (NCT02216409). However, the blockade of the SIRPα-CD47 interaction cannot induce sufficient phagocytic activity and antitumor immune response^18,24^, resulting in failure of the clinical trial (NCT02641002). To improve the response rate of aCD47, researchers have conducted several clinical trials to evaluate the efficacy of aCD47 combined with other anticancer drugs, such as aCD47 with rituximab in treating lymphoma (NTC02953509), azacitidine in treating acute myeloid leukaemia or myelodysplastic syndrome (NTC03248479) and cetuximab in treating advanced colorectal cancer (NTC02953782). To enhance the therapeutic effect of the aCD47 antibody, researchers have employed appropriate combination strategies with additional stimuli, e.g., chemotherapy and phototherapy, to potentiate antitumour immunity^25,26^. Thermal tumour ablation, as a minimally invasive therapy, is one of the most commonly used treatments for a variety of solid tumours, such as hepatic carcinoma, renal carcinoma and osteoma. Hyperthermic ablation is also an alternative technique for patients with failure of chemotherapy or radiotherapy and people who are not good candidates for surgery^27^. In photothermal therapy (PTT), an external light source (usually near infrared light) with strong tissue penetration is absorbed by photothermal nanomaterials for conversion to thermal energy, resulting in cell death by necrosis through increased local temperature^28^. Increasing studies have reported that PTT causes immunogenic cell death, releasing damage-associated molecular patterns^29,30^ that act as endogenous danger signals^31^, hence improving tumour immunogenicity^32^.

Results on graphene have inspired increased research interest in two-dimensional (2D) materials^33–49^. Ultrathin 2D black phosphorus (BP) nanosheets (NSs), as a metal-free photothermal agent that shows a high efficiency for converting NIR to heat, have attracted increased attention in biomedical applications because they are easily biodegradable and biocompatible^50–52^. BP NSs are not stable and are gradually degraded into nontoxic small molecular weight substances such as phosphates and phosphonates^53^. Phosphorus in the form of phosphate participates in almost all cellular processes^54^. For instance, the sugar-phosphate backbone is the main component of the structural framework of DNA and RNA. Living cells need phosphate for transferring cellular energy with ATP. An adult human contains on average ~700 g of phosphorus^55^. Given the excellent biocompatibility properties of BP, it is possible that these ultrathin 2D inorganic NSs have broad potential in the field of biomedical applications as well as in clinical translation.

**Fig. 1:**
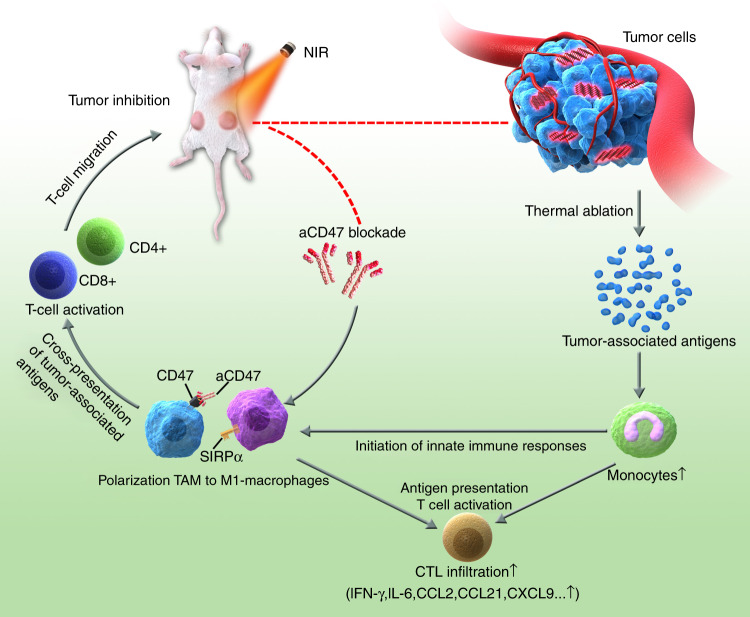
The proposed mechanism of the antitumour immune responses triggered by BP-based photothermtableal effects in combination with aCD47 therapy

Here, we found that BP-based hyperthermic ablation could not only directly destroy tumour cells, releasing tumour-associated antigens and alarmin, but could also act as an effective specific immunological stimulator and boost the poor immune-stimulating properties of tumour antigens, relieving immunosuppression in a tumour microenvironment. BP in combination with immune checkpoint blockade via aCD47 effectively obstructs tumour progression, exerting a significant synergistic effect. We further found that BP plus aCD47 could induce repolarization of TAMs to M1-like macrophages, which not only blocks the CD47/SIRPα interaction, promoting phagocytosis, but also potentiates the local cross-presentation of tumour-associated antigens, thus activating tumour-specific T-cell activity and further inducing the abscopal effect for fighting metastatic cancer (Fig. 1). This finding suggests that photothermal agents might help immune checkpoint blockades overcome tumour immune escape and metastatic progression to achieve a particularly efficient effect in cancer immunotherapy.

## Results

### Characterisation of BPNSs

**Fig. 2:**
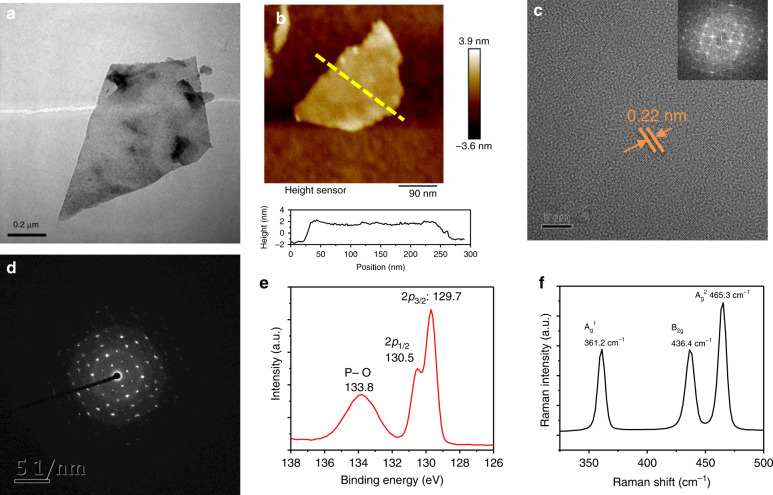
Basic characterisation of BPNSs. **a** TEM image. **b** AFM image. **c** HRTEM image. **d** SAED pattern. **e** XPS curve. **f** Raman curve

Atomic force microscopy (AFM) and transmission electron microscopy (TEM) were used to characterise the morphology of BP NSs. The lateral size of the BPNSs was shown to be 100–200 nm by the TEM image (Fig. 2a). The thickness, as measured by AFM, was observed to be ~3.5 nm (Fig. 2b), which corresponded to approximately six layers, indicating the large aspect ratio of lateral size/thickness. The chemical component of BPNSs was determined by X-ray photoelectron spectroscopy (XPS). Three peaks were observed at 129.7, 130.5 and 133.8 eV. The XPS peaks at 129.7 and 130.5 eV were assigned to 2p3/2 and 2p1/2 in zero-valent BP, and 133.8 eV corresponded to oxidised BP, which indicated the ease of degradability of BP (Fig. 2e). To study the crystallinity of the as-prepared BPNSs, we employed high-resolution TEM, selected-area electron diffraction^56^, fast Fourier transform (FFT), and Raman techniques. The HRTEM image showed clear lattice fringes, with an interatomic distance of 0.22 nm (Fig. 2c), which corresponded to the (014) atomic plane of BP and is consistent with the previous observation^48^. Both the FFT pattern (Fig. 2c) and SAED pattern (Fig. 2d) further confirmed the intact crystalline structure of the BPNSs. The Raman spectrum (Fig. 2f) consisted of three typical Raman peaks, in which 436.4 and 465.3 cm^−1^ were two in-plane phonon modes of B2g and A2g, and 361.2 cm^−1^ was one out-of-plane mode of A1g, again confirming the typical crystal structure of BPNSs. To further enhance the biocompatibility and stability of BPNSs, we used PEG-NH_2_ to functionalise BPNSs. The enhanced stability by PEGylation can be shown by the time-dependent absorbance and photothermal performance (Figs. S1 and S2).

Based on the analyses of the morphological, chemical and crystal structure, we can conclude that the exfoliated BPNSs maintain the corresponding chemical and crystalline state of BP.

### Photothermal effect of BPNSs

**Fig. 3:**
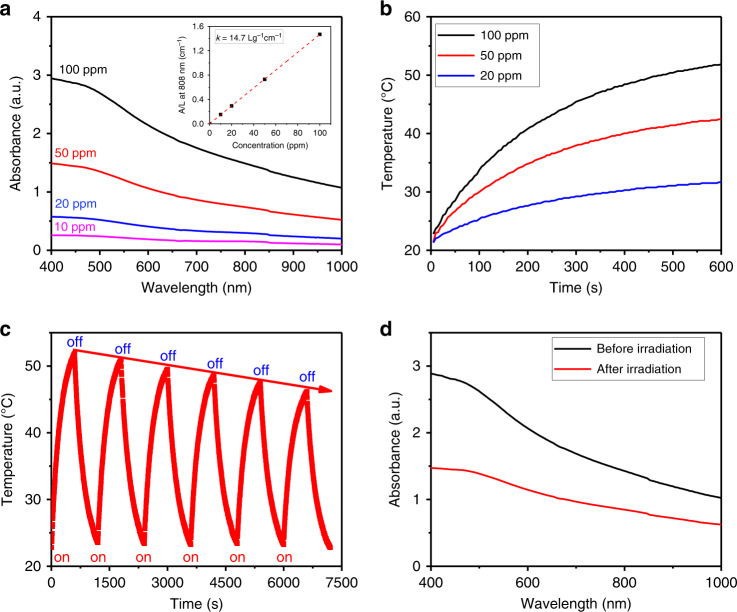
Characterisation of photothermal performance of BPNSs. **a** Absorption of BPNSs. **b** Photothermal temperature increase for different concentrations. **c** The photothermal stability shown by the six photothermal cycles and **d** absorbance before and after six cycles of laser irradiation

Strong absorption is the precondition for high-performance photothermal effects. As shown in Fig. 3a, the BPNSs can realise wide and strong absorption from the UV to NIR region. The extinction coefficient of BPNSs was calculated to be 14.7 Lg^−^^1^ cm^−1^ at 808 nm based on the Beer–Lambert law (Fig. 3a), similar to BP quantum dots^51^. For further characterisation of the photothermal properties of BPNSs, they were dispersed in water at different concentrations, and the dispersion was irradiated with an NIR laser at 808 nm (1.0 W cm^−2^). The temperature gradually increased to a saturation level as the irradiation time increased (Fig. 3b). The photothermal stability of the BPNSs was further evaluated through several photothermal cycles. The BPNS dispersion was first irradiated for 10 min, and then, the irradiation was removed, which gives one photothermal cycle. The photothermal effect of the BPNSs gradually decreased as the number of cycles increased (Fig. 3c), indicating that the BPNSs can be degraded by laser irradiation, which was also proven by the large decrease in absorption after laser irradiation (Fig. 3d). In addition to the strong absorption of BPNSs, their photothermal application can be further highlighted due to their outstanding NIR-induced degradation since they could be cleared from the body after achieving therapeutic effects. The BPNSs gradually degrade after laser irradiation, which decreases their light absorption, resulting in a gradual decrease in temperature.

### Immune stimulatory abilities of BP-based hyperthermic ablation

**Fig. 4:**
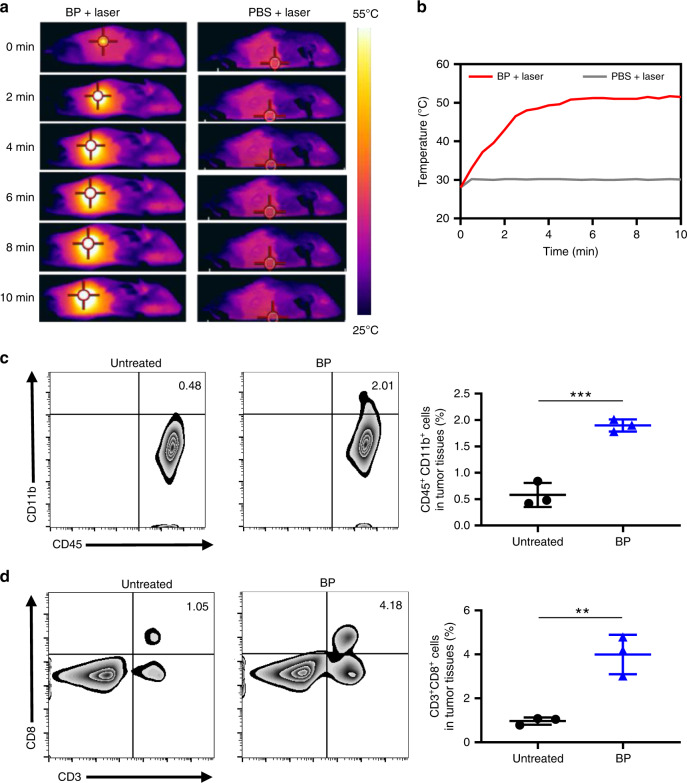
BP-based PTT alone relieves the immunosuppressive tumour microenvironment. **a** IR thermal images of A20 tumour-bearing mice in groups that received two laser irradiation (808 nm, 1 W cm^−2^) treatments for BP and PBS exposure. **b** Temperature change with time at the tumour sites in the BP and PBS groups under 808-nm laser irradiation for 10 min. **c** Representative flow cytometry plot (left) and quantification of murine CD11b^+^ cells on CD45^+^ cells (right). **d** Representative flow cytometry plot (left) and quantification of CD8^+^ T cells on CD3^+^ T cells (right). Data are presented as the mean ± s.e.m. (*n* = 3). The statistical significance was calculated via the two-tailed Student’s *t* test. The asterisk indicates a statistically significant difference (*P < 0.05, **P < 0.01, ***P < 0.001, ****P < 0.001)

Considering the potential ability of photothermal agents to increase tumour immunogenicity, we evaluated whether PTT with BP can trigger a strong immune response to recognise tumour-specific antigens. We first tested the immunological effects of photothermal ablation in BALB/c mice bearing subcutaneous B-cell lymphoma A20 tumours subcutaneously. When the tumour volume reached ~100 mm^3^, the mice were intratumourally exposed to 20 μg of BP with a volume of 50 µl, and the tumours were exposed to 808-nm NIR laser irradiation for 10 min at 1 W cm^−2^. As monitored by a FLIR infrared thermal camera, the temperature of the tumours increased up to ~51 °C after BP and laser treatment; this temperature was high enough to cause significant cell necrosis within the tumour tissues, which can be attributed to a combination of hypoxia, microvascular thrombosis and ischaemia (Fig. 4a, b)^57^. When the tumour’s temperature is higher than 50 °C, the surface of the tumour tissue can be observed to be burning after 48 h of laser irradiation, and it is predicted that the tumour tissue can also be destroyed. In addition, the body temperature returned to normal 3–5 min after laser irradiation. However, for the tumours injected with phosphate-buffered solution (PBS) under laser irradiation, the temperature was maintained at ~30 °C, which was insufficient to induce any tissue damage^57^.

To determine the status of immune responses in the tumours after BP-based photothermal treatment, we sacrificed the mice, and the tumour cells from the BP-treated and control groups were collected at 48 h after the light irradiation treatment and costained with CD45/CD11b and CD3/CD8 antibodies for flow cytometry analysis. After photothermal ablation of the tumours by BP NSs, a dramatic increase in murine monocytes (CD45^+^CD11b^+^), the direct precursors of macrophages and dendritic cells, within the tumours was observed compared to that in the control group, suggesting that more monocytes may be recruited to the ablated tumour tissues for phagocytosis and presentation of tumour-specific antigens to initiate and regulate immune responses (Fig. 4c)^58^. In addition, more CTL (CD3^+^CD8^+^), which play a key role in promoting the apoptotic death of cancer cells^59^, infiltrated the tumour after BP treatment with NIR irradiation than that in the control group (Fig. 4d). Consequently, after tumour tissue destruction by hyperthermic ablation by BP, monocytes may be activated to initiate innate immune responses, and then, the presentation of tumour-specific antigens released by photothermal treatment triggered CTL-mediated adaptive immunity. That is, in addition to directly destroying tumour cells, BP-based PTT could also serve as an effective immune stimulator, simultaneously reversing the tumour immunosuppressive microenvironment and promoting the CTL-mediated antitumour immunological effect. Overall, this phenomenon provides direct evidence that BP-based hyperthermic ablation destroys tumours and activates both innate and adaptive antitumour immune responses. Hence, we hypothesise that BP with a photothermal effect acting as a specific immunological stimulator may be promising for enhancing cancer immunotherapy.

### BP-based PTT and aCD47 combined treatment in vivo

**Fig. 5:**
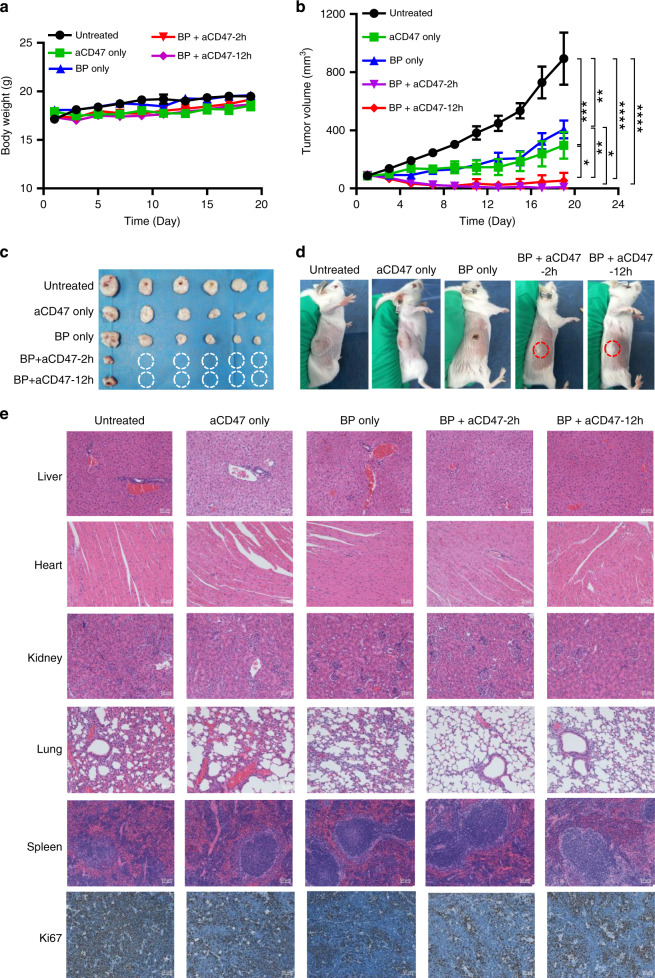
The antitumour effect of BP-based PTT plus aCD47 therapy. **a** Body weight changes of the mice for different treatments. **b** Tumour growth kinetics in various treatment groups. **c** Tumours collected after treatments. **d** Typical photographs of the mice after treatments. **e** H&E staining of major organs and Ki-67 IHC staining of tumours at 19 days after various treatments. Data are presented as the mean ± s.e.m. (*n* = 6). The asterisk indicates a significant difference calculated by one-way ANOVA (*P < 0.05, **P < 0.01, ***P < 0.001, ****P < 0.001)

Most immune checkpoint blockades exert limited antitumour efficacy due to CTL exhaustion within the tumour microenvironment. Recently, the aCD47 antibody, which targets the ‘don’t eat me’ signal, has been proven to be a highly promising agent for tumour immunotherapy^60^. CD47 is overexpressed in various cancers, such as acute lymphocytic leukaemia, myeloma, non-Hodgkin’s lymphoma and lung cancer. Here, we used the B-cell lymphoma cell (A20) model to evaluate the antitumour effect of aCD47 or BP plus aCD47 in immunotherapy research. The A20 cell lines are B-cell lymphoma lines and have histological features of lymphoma. Subcutaneous implantation of A20 requires a large number of cells to form a subcutaneous tumour after 10 days. In addition, subcutaneous inoculation facilitates intratumoural injection and measurement of tumours. Encouraged by the excellent immunological effect promoted by BP hyperthermic ablation, we thus hypothesised that such a specific immunity stimulator may produce a synergistic enhancement effect with the aCD47 checkpoint inhibitor. Next, to test the synergistic antitumour efficacy of BP-based PTT with aCD47, we subcutaneously inoculated 5 × 10^6^ B-cell lymphoma cells (A20) in the right flank of Balb/c mice. Ten days later, when the tumour volume reached ~100 mm^3^, the Balb/c mice were randomly assigned to five groups: untreated (group 1), BP with 808-nm NIR irradiation (group 2, 20 µg of BP per mouse), aCD47 (group 3, 50 µg of aCD47 per mouse), BP with 808-nm NIR irradiation plus aCD47 (group 4, 20 µg of BP and 50 µg of aCD47 per mouse at 2-h post PPT treatment) and BP with 808-nm NIR irradiation plus aCD47 (group 5, 20 µg of BP and 50 µg of aCD47 per mouse at 12-h post PPT treatment). The mice in groups 3, 4 and 5 were injected with aCD47 at a dose of 50 µg per mouse on day 5 for the second time. Body weight and tumour growth were then monitored in the following 3 weeks. There was no obvious influence on the body weights of the mice during the treatments (Fig. 5a). For the tumours of the mice treated with BP-based PTT (group 2) or aCD47 alone (group 3), the speed of tumour growth was partially delayed compared to that in the untreated group (group 1) (Fig. 5b). Combination therapy with BP-based hyperthermic ablation plus aCD47 (groups 4 and 5) significantly suppressed tumour growth, as five of six mice receiving the combined treatment were macroscopically tumour free until finishing the experiment on day 19 (Fig. 5b–d). Notably, compared to those in group 5, the tumours in group 4 showed the slowest growth speed at the end of the experiment, although this difference was not significant, implying that the time point of aCD47 administration may be a key factor for effective therapeutic efficacy.

To further investigate whether various treatments cause pathological changes, we collected tissues from major organs, including heart, lung, liver, spleen and kidneys, as well as tumours from different groups of mice at 19 days post treatment, and haematoxylin and eosin (H&E) staining was performed. The histopathological analysis of lungs showed the presence of inflammation, hyperaemia, reduced alveolar volume and alveolar hyperplasia in the control groups (groups 1, 2 and 3), while the BP and aCD47 combined treatments (groups 4 and 5) resulted in reduced pathological abnormalities. Similarly, prominent oedema in the liver was observed in the control groups (groups 1, 2 and 3), but much less oedema was observed in the combination treatment groups (groups 4 and 5). In addition, lymphoma cell metastasis distributed in the spleen was observed in the control groups, whereas limited spleen metastasis was observed in the combination-treated groups. The above results suggest that BP and aCD47 combination treatments resulted in inhibition of spleen metastasis and promoted pathological damage in the A20 tumours. No obvious inflammation, lesions or histological changes were observed in the heart and kidneys in each group, indicating that various treatments cause low toxicity to the heart and kidney (Fig. 5e). However, severe necrosis of tumour cells, including deformed cells and even nuclear condensation and fragmentation, was observed from the tumour slices of the BP-based hyperthermic ablation plus aCD47 groups (groups 4 and 5), while only partial cell destruction was found in the BP with 808-nm NIR irradiation group (group 2). Since Ki-67 is a cellular marker strongly associated with proliferation and Ki-67-positive tumour cells are often related to cancer cell proliferation and growth^61^, tumour slices sampled on day 19 after the different treatments were subjected to immunohistochemical staining with the Ki-67 antibody for analysis of the proliferative activities. The results showed that the expression of Ki-67 was observably reduced in the tumour tissues of the BP + aCD47 combined treatment groups, and slight suppression of Ki-67 expression was detected in the BP with 808-nm NIR irradiation group, which was consistent with the H&E results, confirming the inhibition of proliferation and growth of cancer cells by the photothermal ablation effect of BP as well as the BP plus aCD47 combination treatments (Fig. 5e). In general, the therapeutic effect of BP-based hyperthermic ablation in combination with aCD47 was substantially stronger than that of the groups treated with BP-based PPT or aCD47 only, demonstrating the significant synergistic antitumour effect of the combination treatment.

### Immune responses after BP-PTT plus aCD47 blockade

**Fig. 6:**
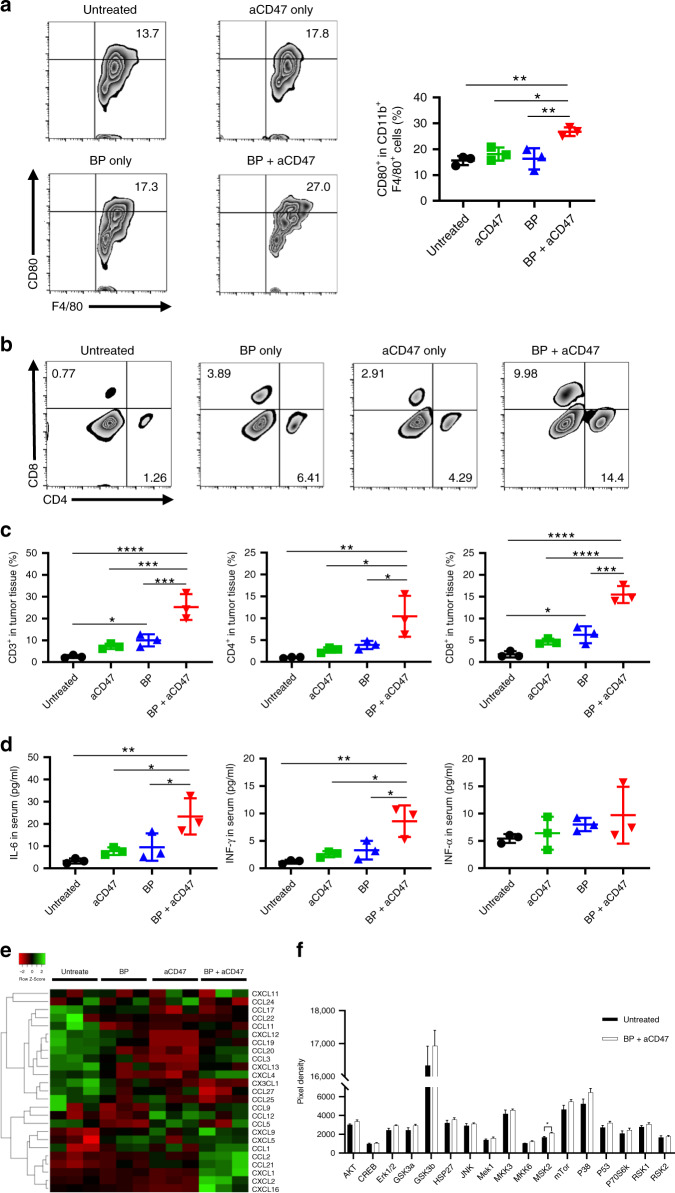
BP + aCD47 therapy triggers antitumour innate and adaptive immune responses. **a** Representative flow cytometry results (left) and quantification of CD80^+^ cells gating on CD11b^+^F4/80^+^ cells (right). **b** Representative flow cytometry results of T-cell infiltration. **c** Absolute percentage of CD8^+^, CD3^+^ and CD4^+^ T cells within the tumours following different treatments. **d** Cytokine levels in the serum isolated from mice on day 5 after various treatments. **e** Chemokine levels in the tumour tissues from mice isolated at 48 h after various treatments. **f** Quantification of MAPK phosphorylation proteins within the tumours at 48-h post treatment. Data are presented as the mean ± s.e.m. (*n* = 3). The asterisk indicates a significant difference calculated by one-way ANOVA (*P < 0.05, **P < 0.01, ***P < 0.001, ****P < 0.001)

Accumulating evidence has shown that aCD47 antibodies target tumour-specific macrophages by re-education of the M2 phenotype to M1-like macrophages for inhibition of tumour progression^62,63^. To examine the synergistic anticancer activity of a combination of BP-based PTT and aCD47 in more detail, we randomly allocated A20 tumour-bearing mice into four groups: group 1: untreated; group 2: BP with 808-nm NIR irradiation (20 µg of BP per mouse); group 3: aCD47 (50 µg of aCD47 per mouse); group 4: BP with 808-nm NIR irradiation plus aCD47 (20 µg of BP and 50 µg of aCD47 per mouse at 2-h post PPT treatment). The residual tumour tissues from these groups were harvested at 48-h post treatment. The M1-like macrophages in the tumour tissues were analysed by flow cytometry. The percentage of M1-type (CD80^+^CD11b^+^F4/80^+^) macrophages infiltrated in the residual tumours from the BP-based PPT and aCD47 combination treatment group was significantly higher than that in the control groups, suggesting that this combined therapy induced the polarisation of tumour-specific macrophages towards the M1 phenotype and thus altered the immunosuppressive status into a more permissive tumour microenvironment to promote tumour regression (Fig. 6a). T cells have a vital role in the immune response and fight against cancers in both direct and indirect ways^64^. Cytotoxic CD8^+^ T cells (CD8^+^) can find cancer cells and then be stimulated to kill them directly^65^, while helper CD4^+^ T cells (CD4^+^) fight cancer cells indirectly by activating macrophages and cytotoxic T cells and secreting cytokines that regulate the immune response^66^. To further investigate whether this combination treatment boosts tumour-specific T-cell responses, we tested different T-cell populations in tumours after various treatments. Consistent with the above results, the absolute numbers of tumour-infiltrating lymphocytes (TILs, CD3^+^) and CD8^+^ T cells were increased in the BP-based PPT group compared to the control group, suggesting robust TIL and CD8^+^ T-cell infiltration by hyperthermic ablation (Fig. 6b, c). We also found that the proliferation of CD3^+^ cells was dramatically increased with the combination treatment of BP + aCD47 (Fig. 6c). Moreover, the infiltration numbers of CD8^+^ and CD4^+^ cells were also significantly enhanced in the tumours after the BP + aCD47 combination treatment compared to the control (Fig. 6b, c), thus triggering efficacious adaptive antitumour immunity.

Cytokines secreted by both innate and adaptive immune cells also have an important role in adjusting the immune response^67^. Sera of mice bearing A20 tumours were sampled at 120-h post treatment for analysis of cytokines, including IL-6, TNF-α and IFN-γ. Our results showed that BP and aCD47 combined treatment could upregulate the secretion levels of IFN-γ, which is a key activator of macrophages and is released predominantly by natural killer cells, CD4^+^ and CD8^+^ T cells once antigen-specific immunity is induced^68^. Similarly, the levels of IL-6 that can stimulate the proliferation of lymphocytes and further shift the T-cell immune response from suppression to responsiveness^69^ were also strongly increased after the BP and aCD47 combined treatment compared to the controls. The enhanced secretion of IL-6 and IFN-γ confirmed the promotion of the innate immune response along with CTL-mediated immunity by the BP and aCD47 combination treatment. Nevertheless, a nonsignificant increase in TNF-α, which can be a tumour promoter during growth, invasion and metastasis as well as a cancer killer with cytotoxic and immunoregulatory effects^70^, after various treatments indicated that the role of this multifunctional cytokine was ambiguous (Fig. 6d).

Chemokines are small cytokines or signalling proteins known as chemotactic cytokines that can induce directional chemotaxis in nearby responding cells^71^. In the tumour microenvironment, chemokines can modulate tumour invasion, proliferation and metastasis. Since chemokines are involved in the recruitment of immune cells such as monocytes, macrophages, mast cells, eosinophils, neutrophils and T lymphocytes, they directly and indirectly impact the immune response to tumours, affecting the progression of tumours and the anticancer therapeutic effect^72^. To further investigate this phenomenon, we collected mouse tumour tissues after various treatments for 48 h and subjected them to chemokine quantification using a mouse chemokine array. The combination treatment caused increased secretion of CCL2, CCL21, CXCL1, CXCL2, CXCL5, CXCL9 and CXCL16, whereas the levels of CCL11, CCL17, CCL19, CCL22, CCL25, CCL27 and CX3CL1, which are associated with protumourigenic effects, were decreased in the tumours (Fig. 6e, Table S1, Supporting information). In particular, CCL21 chemoattracting dendritic cells and T lymphocytes are critical in initiating a T-cell response^73^, while CCL2 attracts monocytes or macrophages and CXCL9 recruits T cells and NK cells^74^, confirming the moderating effect on immunosuppression in the tumour microenvironment induced by the combination treatment of BP and aCD47.

The mitogen-activated protein kinase (MAPK) signalling pathway is important for the regulation of numerous cellular processes. Dysregulation of MAPK signalling may result in tumour occurrence^75^. To determine whether the MAPK signalling pathway has a role in the BP + aCD47 combined treatment for inhibition of cancer growth, we collected tumour tissues at 48-h post treatment from the untreated and combination groups and analysed them by a MAPK phosphorylation array. The results showed that only the expression level of mitogen- and stress-activated kinase 2 (MSK2), which is activated downstream of the p38 MAPK pathways, was significantly higher compared with that of the control (Fig. 6f, Table S2, Supporting information). MSKs have been shown to modulate the production of anti-inflammatory cytokines by controlling their transcription in macrophages and dendritic cells and promote cell death signalling through other pathways^76^, revealing that the BP and aCD47 combined treatment effectively inhibits tumour growth, probably involving MSK2 activation. Further studies are needed to unravel the exact role of MSK2 in the BP and aCD47 combination treatment against cancer. Above all, our results demonstrate that the combination therapy of BP-PTT and aCD47 could simultaneously promote effective phagocytosis of tumour cells by M1-like macrophages and the activity of tumour-associated T cells, moderating the inherently nonimmunogenic property of tumours.

### BP in combination with aCD47 for inhibition of distal tumours

**Fig. 7:**
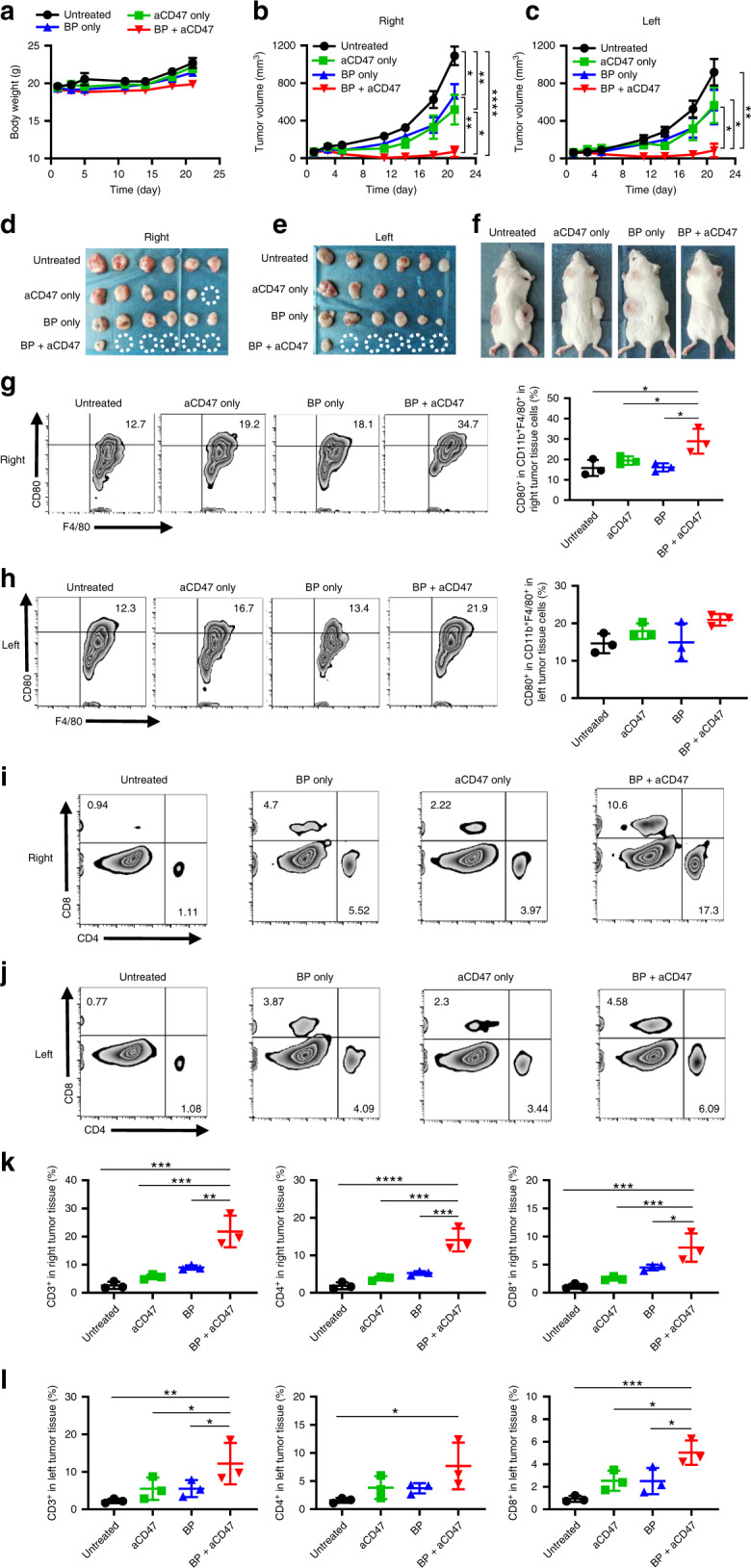
Local treatment of BP + aCD47 for systemic antitumour effects. **a** Body weight changes in the mice after different treatments. Tumour growth curves for the right (**b**) and left (**c**) tumours in different groups after various treatments. **d, e** Photographs of the right and left tumours in different groups collected at day 21. **f** Representative photographs of tumour-bearing mice at day 21. Representative flow cytometry plot and quantification of CD80^+^ cells gating on CD11b^+^F4/80^+^ cells in the right (**g**) and left (**h**) tumours. Representative flow cytometry plot of T-cell infiltration in the right (**i**) and left (**j**) tumours. Absolute percentage of CD3^+^, CD4^+^ and CD8^+^ T cells in the right (**k**) and left (**l**) tumours following different treatments. Data are presented as the mean ± s.e.m. (*n* = 6). The asterisk indicates a significant difference calculated by one-way ANOVA (*P < 0.05, **P < 0.01, ***P < 0.001, ****P < 0.001)

It has been reported that ~90% of cancer deaths are caused by metastasis^77^. Since BP in combination with aCD47 therapy has the ability to trigger immune responses, we further examined the impact of combined treatment on a model of distant metastasis to determine whether local treatment could promote systemic immunity against cancer. A20 cancer cells were subcutaneously transplanted into both the left and right flanks of Balb/c mice to mimic metastatic cancer. The tumours on the right and left sides were defined as the primary and distal tumours, respectively. When the tumour volume reached ~100 mm^3^, the A20 tumour-bearing mice were randomly allocated into four groups as discussed in ‘Immune responses after BP-PTT plus aCD47 blockade’. First, the primary tumours were irradiated with an NIR laser (1 W cm^−2^, 10 min) for groups 2 and 4. Subsequently, the mice in groups 3 and 4 were intratumourally injected with 50 µg of aCD47 at day 1 and day 5, whereas the left distal tumours were left untreated. As expected, the growth of primary tumours was inhibited by BP-based PTT and aCD47 treatment, but the distal tumours on the left side without treatment also showed pronounced regression, and five of the animals were macroscopically tumour free on day 21, achieving better efficacies than those of the other control groups (Fig. 7b–f). The body weights of the mice remained unaffected during the treatment process (Fig. 7a). Accordingly, BP-based PTT in combination with aCD47 could not only directly destroy the primary tumours but also significantly suppress the distal tumours without any treatment, indicating the potential antimetastatic ability of this combination strategy.

To further investigate this phenomenon, we collected immune cells in both the primary and distal tumours for flow cytometric analysis. In accordance with the previous results, the levels of M1-type macrophages were significantly increased in the primary tumours treated with BP plus aCD47 (Fig. 7g, h). In contrast, the percentages of M1-like macrophages in the distant metastases showed no difference in all four groups, suggesting that the strong suppressive effect on distant tumour growth was not directly attributed to polarisation of TAMs. Furthermore, we examined the subgroups of T cells in tumours. Interestingly, in both primary and distant tumours, the infiltration levels of CD3^+^, CD4^+^ and CD8^+^ T cells were substantially higher in the BP plus aCD47 combination treatment group than in the control groups (Fig. 7i–l), revealing the adaptive immunological responses triggered in distant metastases as well. These results indicate that although BP hyperthermic ablation acts as a specific immunological stimulator to improve tumour immunogenicity and promote the CTL-mediated antitumour immunological effect, a combination with aCD47 blockade could potentiate the local cross-presentation of tumour-specific antigens by macrophages, boost tumour-associated T-cell responses and further induce abscopal effects, thus stimulating the systemic anticancer immune response.

## Discussion

In summary, BP-based PTT with photothermal effects could not only directly destroy or even kill tumour cells but could also recruit increased levels of monocytes to ablated tumour tissues for the initiation of innate immune responses and release tumour-specific antigens from necrotic cancer cells to trigger CTL-mediated adaptive immunity, thus serving as an effective specific immunological stimulator that improves the inherently poor immunogenicity of tumours. BP in combination with aCD47 strongly suppressed the proliferation and growth of cancer cells, exerting a significant synergistically enhanced antitumour effect. Moreover, BP-based hyperthermic ablation plus aCD47 treatment induced the polarisation of tumour-specific macrophages towards the M1 phenotype, blocking the ‘don’t eat me’ signal of CD47-SIRPα in tumour cells and thus promoting phagocytosis by macrophages. Activated macrophages may potentiate the local cross-presentation of tumour-specific antigens and then facilitate the production of tumour antigen-specific T cells that may migrate into other distant tumours to destroy cancer cells that express the same tumour-specific antigens, thus resulting in induction of the abscopal effect and showing promise for inhibition of metastatic cancers (Fig. 1). In short, BP-based photothermal effects moderated immunosuppression in the tumour microenvironment, which further enhanced antitumour immunity to facilitate aCD47 blockade cancer immunotherapy. The innate and adaptive immunity activated by BP plus aCD47 combined therapy promoted the local and systemic anticancer immune responses. Our combination strategy provides a promising platform to enhance the therapeutic efficacy of aCD47 in solid tumours. Given the excellent biodegradability property of BP, it is possible that these inorganic NSs have potential in biomedical applications and clinical translation for combination with cancer immunotherapy to induce synergistic effects^78^.

## Materials and methods

### Materials

Bulk BP was purchased from Macklin Company (Shanghai, China). PEG-NH2 (5000 Da) was purchased from Nanocs, Inc. (New York, USA). Other reagents were analytical grade. Ultrapure water (25 °C, 18.25 MΩ cm) was used for water-based dispersions.

### Fabrication of BPNSs and PEG-modified BPNSs

Liquid-phase exfoliation was used to exfoliate BP. Typical exfoliation was mainly divided into three steps: grinding, probe sonication and bath sonication. One hundred milligrams of bulk BP was dispersed in 1-methyl-2-pyrrolidone (NMP) (10 ml). Then, the bulk BP was ground by a hammer in NMP. After grinding, 90 ml of NMP was further added to the mixture. Then, the mixture underwent probe sonication with a power of 260 W for 8 h. To avoid overheating produced by probe sonication, we set the sonication process to an interval mode with an on/off cycle of 4/4 s, and the BP dispersion was immersed in ice water. After probe sonication, the BP dispersion underwent bath sonication for 12 h at a power of 300 W. The bath temperature was set to 10 °C.

After these processes, appropriate BPNSs can be obtained and need to be selected from the mixture with bulk BP. Therefore, the resulting suspensions were centrifuged at 7000 rpm to remove the large BPNSs and bulk BP. The supernatant with the expected BPNSs was collected for further centrifugation at 17000 rpm for 30 min. Then, the precipitate was gathered and dried in a vacuum drying chamber. Then BPNSs were sealed in tubes and protected by the tinfoil package to avoid degradation. Then, the samples were stored in the refrigerator at 4 °C for further use.

For fabrication of PEG-modified BPNSs, PEG-NH_2_ was mixed with BPNSs at a mass ratio of 2:1. After the sample was stirred for 2 h, the resulting dispersion was centrifuged through Amicon tubes (MWCO 100 kDa; Millipore) at 3000 rpm (4 °C) for ~20 min to remove the uncoated PEG-NH2 molecules and was further washed two times using the same centrifugation method. The pure PEG-modified BPNS sample was resuspended in PBS and stored in a 4 °C refrigerator for further use.

### Characterisations

TEM (JEM1230) was used to measure the lateral size of the BPNSs, and AFM (Bruker, Dimension Fastscan) was used to measure the thickness. For the TEM sample, the BPNS dispersion was placed on a copper grid and dried in an oven. The spin-coating method was used to prepare the AFM sample on a silicon substrate. Then, the high-resolution AFM images were scanned at 512 pixels in one line. HRTEM and SAED were performed on Tecnai G2 F30 equipment with a voltage of 300 kV. The XPS spectrum was acquired through a ULVAC PHI 5000 Versa Probe II with Al Kα (*hυ* = 1486.7 eV, *λ* = 0.83 nm). Raman measurements were made on a HORIBA modular Raman spectrometer with 514-nm laser excitation. The UV–vis absorption spectrum was measured at nm employing a Cary 60 spectrometer from Agilent Company. A semiconductor diode laser, LSR808H (Lasever, Inc.), was used as the excitation source for conducting photothermal experiments. The measurement of photothermal experiments was carried out by using both a thermocouple and a thermal imaging camera (FLIR E-75).

### Cytokine detection

Levels of IFN-γ, IL-6, TNF-α in serum isolated from blood obtained by heart punctures of the mice on day 5 after different treatments were quantified through enzyme-linked immunosorbent assay kits (IFN-γ, R&D, cat. no. MIF0; TNF-α, R&D, cat. no. MTA00B; IL-6, R&D, cat. no. M6000B), following the manufacturer’s instructions. All animal experiments were conducted following protocols approved by the Shenzhen International Institute of biomedical Research complied with all relevant ethical requirements.

### MAPK phosphorylated protein detection

At 48 h after no treatment or the BP + aCD47 combined treatment, tumour slices were sampled and homogenised in the presence of cell lysis buffer, proteinase inhibitor and phosphatase inhibitor cocktail. The supernatants were collected, and phosphorylated proteins related to the MAPK pathway were analysed by a Human/Mouse MAPK Phosphorylation Array (RayBiotech, cat. No. AAH-MAPK-1-8), following the manufacturer’s instructions.

### Flow cytometry

For determination of the immunological effect of BP-aCD47 combination treatment, A20 tumour-bearing mice with a volume of ~100 mm^3^ were randomly assigned to four groups and treated as described previously. At 48-h post treatment, the tumour slices were collected from the sacrificed mice for detection of the immune effect. Briefly, tumour slices were cut into small pieces and subjected to digestion by collagenase type IV (Sigma-Aldrich, cat. no. C5138), hyaluronidase (Sigma-Aldrich, cat. no. H3506) and DNase I (Sigma-Aldrich, cat. no. 10104159001) at 37 °C for 30 min with shaking. RBC lysis buffer (Biolegend, cat. no. 420301) was used for the removal of red blood cells. Dead versus live cells were measured by a Zombie NIR^TM^ Fixable Viability Kit (Biolegend, cat. no. 423106), following the manufacturer’s instructions. The cells were stained by antibodies with fluorescence-labelled anti-CD3-APC (Biolegend, cat. no. 100236, clone 17A2), anti-CD8-FITC (Biolegend, cat. no. 100706, clone 53-6.7), anti-CD4-PE (Biolegend, cat. no. 100407, clone GK1.5), anti-CD45-APC (Biolegend, cat. no. 103112, clone 30-F11), anti-F4/80-FITC (Biolegend, cat. no. 123107, clone BM8), anti-CD11b-PE/Cy7 (Biolegend, cat. no. 101216, clone M1/70) and anti-CD80-PE (Biolegend, cat. no. 104708, clone 16-10A1), following the manufacturer’s instructions. CD3^+^CD8^+^ cells were defined as CTL. F4/80^+^CD11b^+^CD80^+^ cells were defined as M1 phenotype macrophages, and CD45^+^CD11b^+^ cells were defined as murine monocytes. The stained cells were measured on a DxFLEXF flow cytometer (Beckman Coulter), and data were analysed using FlowJo V10 software.

### Statistical analysis

Mean and standard error of the mean (s.e.m.) were used to show the data. Statistical analyses were performed using two-tailed unpaired Student’s *t* test for the comparisons of two independent groups, while one-way analysis of variance was used for comparisons of more than two groups by using GraphPad Prism software, and *P* < 0.05 (*), *P* < 0.01 (**), *P* < 0.001 (***) and *P* < 0.0001 (****) denote statistical significance.

Supplementary information accompanies the manuscript on the Light: Science and Applications website (http://www.nature.com/lsa).

## Supplementary information

Supplementary Information
