# Supplementary material for: Black phosphorus-based photothermal therapy with aCD47-mediated immune checkpoint blockade for enhanced cancer immunotherapy
Source: Light Sci Appl. 2020 Sep 15;9:161. doi: 10.1038/s41377-020-00388-3 (PMC7492464; doi:10.1038/s41377-020-00388-3)
Supplement: Supplementary file 1 — Supplementary Information [file 41377_2020_388_MOESM1_ESM.docx]

**Supplementary Information for**

**Black phosphorus-based photothermal therapy with aCD47-mediated immune checkpoint blockade aCD47 for enhanced cancer immunotherapy**

Zhongjian Xie^a,b,1^, Minhua Peng^b,e,1^, Ruitao Lu^d,1^, Xiangying Meng^b^ , Weiyuan Liang^a^, Zhongjun Li^a^, Bin Zhang^a^, Guohui Nie^a^, Ni Xie^a^, Han Zhang^a,^* and Paras N. Prasad*^,c^

^a^Key Laboratory of Optoelectronic Devices and Systems of Ministry of Education and Guangdong Province, Institute of Microscale Optoelectronics, and Otolaryngology Department and Biobank of the First Affiliated Hospital, Shenzhen Second People's Hospital, Health Science Center, Shenzhen University, Shenzhen 518060, P. R. China

^b^Shenzhen International Institute for Biomedical Research, 518116 Shenzhen, Guangdong, China

^c^Institute for Lasers, Photonics, and Biophotonics and Department of Chemistry, University at Buffalo, State University of New York, Buffalo, USA

^d^School of Basic Medical Sciences, Guangzhou Medical University, 511436, Guangzhou, Guangdong, China

^e^Shenzhen Institutes of Advanced Technology, Chinese Academy of Sciences, 518055 Shenzhen, Guangdong, China

^1^M.P. and R.L. contributed equally to this work.

*corresponding author

E-mail address: [hzhang@szu.edu.cn](mailto:hzhang@szu.edu.cn) (H. Zhang); [pnprasad@buffalo.edu](mailto:pnprasad@buffalo.edu) (P. Prasad)

**Stability**

The BP and PEGylated BP (BP-PEG) were dispersed in water and PBS respectively (Figure S1). For either BP or BP-PEG in water, they show negligible agglomeration. For PBS dispersions, it was observed that BP-PEG can obtain an enhanced stability than the pure BP dispersion over 24 h. However, in 48 h, both the BP and BP-PEG in PBS dispersions almostly aggregated and deposited.

The influence of agglomeration on photothermal performance was also investigated (Figure S2). For all dispersions, the maximum photothermal temperature decreased as time went on. Compared with the BP dispersion in PBS, the BP-PEG dispersion in PBS showed a slower decreasing speed of photothermal temperature, indicating the enhanced stability as revealed by absorbance.


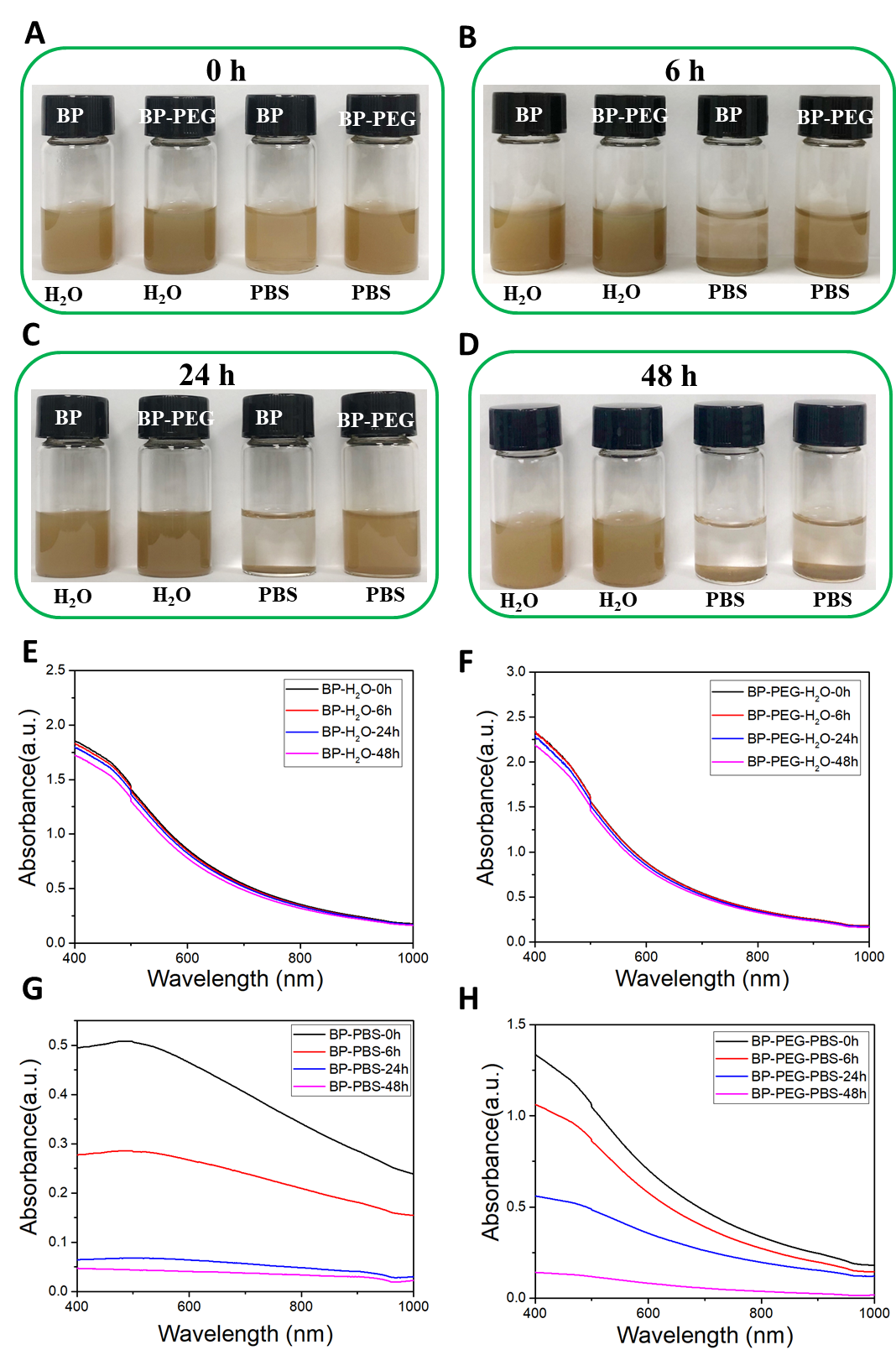


Figure S1. Time-dependent BP dispersions in water and PBS. (A-D) The photographs and (E-H) absorbance of the dispersions at different time points.


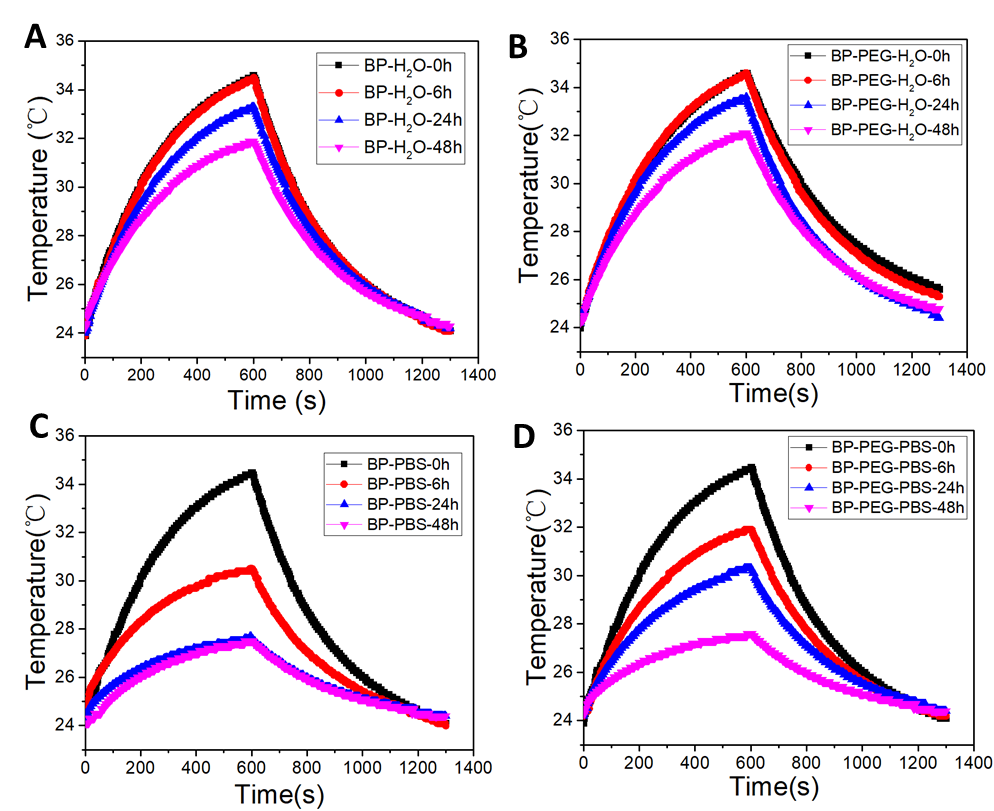


Figure S2. The photothermal performance at different time points for (A) BP in water; (B) BP-PEG in water; (C) BP in PBS; (D)BP-PEG in PBS.

**In vivo tumor treatment.**

To investigate the antitumor effect of BP and aCD47 combined treatment, 5×10^6^ of A20 cells were dispersed in 100 µl of PBS and then were inoculated in the right flank of balb/c mice subcutaneously. When the tumor grew to approximately 100 mm^3^, the mice were randomly divided into five groups randomly (n=6, per group): (1) untreated: intratumorally injected with 50 µl of PBS, (2) BP only: intratumorally injection with 20 µg of BPNSs and subsequently exposed to irradiation using an 808 nm NIR laser at 1 W cm^-2^ for 10 min, (3) aCD47 only: intratumorally injected with 50 µg of aCD47 only on day 1 and day 5, (4) BP+aCD47-2h: intratumorally injected with 20 µg of BP nanoparticles and irradiated with an 808 nm laser (1 W cm^-2^, 10 min), and further treated with 50 µg of aCD47 at 2h and on the 5^th^ day after BP treatment. (5) BP+aCD47-12h: intratumorally injection with 20 µg of BPNSs and exposed to an 808 nm laser (1 W cm^-2^, 10 min), and then treated with 50 µg of aCD47 at 12h and on the 5^th^ day after BP treatment. The temperature changes on the tumor’s surface were measured by an IR thermal camera (FLIR system). Through measuring length and width, the tumor sizes were calculated based on the following formula: length × width^2^ × 0.5. On 19 days post treatment, the tissues of main organs including heart, liver, lung, kidney, and spleen as well as the tumor from the above five groups were surgically excised, fixed in 4% paraformaldehyde and subsequently stained by hematoxylin and eosin (H&E) for histology and cytology analysis following the standard protocols. In the meantime, tumor slices collected and fixed in 4% paraformaldehyde, were subjected to immunohistochemical staining with Ki-67 (D3B5) Rabbit mAb (dilution 1:400, mouse preferred, CST, cat. No. 12202S) according to the standard protocols.

For a bilateral tumor model, 5×10^6^ of A20 cell suspensions were transplanted into the right (primary tumor) and left flank (distant tumor) of mice. Ten days later, tumors in the right flank were treated with (1) 50 µl of PBS, (2) BP only (20 µg per mouse, with laser irradiation), (3) aCD47 only (50 µg) and (4) BP+aCD47-2h (20 µg of BP with laser irradiation and 50 µg of aCD47), as described above. Subsequently, the primary tumors were subjected to an infrared laser irradiation (808 nm, 1 W cm^-2^, 10 min), and injected with 50 µg of aCD47 at two hours and at 5 days post irradiation. Primary and distant tumor sizes as well as body weight were monitored.

**Antibody, cell lines and animals.**

aCD47 was bought from Biolegend (Cat. No. 127519, clone miap301). A20 cells (B cell lymphoma line, ATCC) were cultured in a RPMI 1640 medium (Gibco, (Cat. No. C11875500BT) containing 10% fetal bovine serum (CellMax Cat. No. SA301.02), 1% streptomycin/penicillin (Gibco, Cat. No. 15140-122), 5% CO_2_ at 37 ^o^C in an incubator. 5-6 weeks female balb/c mice were bought from the Beijing Vital River Laboratory Animal Technology Co., Ltd.

**Chemokine detection.**

At 48 hours post treatment, mouse tumor tissues were harvested and then lysed with cell lysis buffer in the help of protease inhibitor cocktail. Supernatants were collected and concentrations of different chemokines were measured by the Mouse Chemokine Array (Raybiotech, cat. No. QAM-CHE-1-1) according to the manufacturer’s instruction (Table S1, S2).

**Table S1**. Individual pixel density of mouse chemokines in the tumour tissues from mice isolated

at 48 h (n=3).

| Chemokine name | Untreated | | | BP only | | | aCD47 only | | | | BP+aCD47 | | | |
| --- | --- | --- | --- | --- | --- | --- | --- | --- | --- | --- | --- | --- | --- | --- |
|  | 1 | 2 | 3 | 1 | 2 | 3 | 1 | 2 | 3 | 1 | | 2 | 3 |  |
| CCL21 | 817 | 732 | 866 | 908 | 1108 | 939 | 1175 | 1171 | 1073 | 1315 | | 1384 | 1620 |  |
| CXCL13 | 11364 | 11228 | 13461 | 7802 | 3691 | 9284 | 5579 | 3675 | 3518 | 7559 | | 7707 | 5661 |  |
| CCL27 | 1891 | 1822 | 2029 | 1833 | 1586 | 1735 | 1729 | 1629 | 1830 | 1352 | | 1445 | 1723 |  |
| CXCL16 | 15399 | 16562 | 15358 | 14532 | 14113 | 11670 | 10147 | 10222 | 10306 | 35540 | | 25527 | 19560 |  |
| CCL11 | 61512 | 93396 | 69690 | 22864 | 34548 | 43918 | 37002 | 37649 | 48014 | 53516 | | 49984 | 35092 |  |
| CCL24 | 20519 | 27646 | 39860 | 22244 | 28239 | 36289 | 22714 | 33006 | 42748 | 21163 | | 26533 | 15171 |  |
| CX3CL1 | 1997 | 2284 | 2620 | 1926 | 1444 | 1902 | 1501 | 1578 | 1319 | 1017 | | 1306 | 1261 |  |
| CXCL11 | 3868 | 3367 | 3958 | 3902 | 3348 | 3780 | 3377 | 4251 | 3783 | 3163 | | 4538 | 4102 |  |
| CXCL1 | 244 | 226 | 690 | 278 | 518 | 597 | 64 | 190 | 317 | 2533 | | 2315 | 3551 |  |
| CXCL5 | 492 | 460 | 342 | 533 | 578 | 599 | 558 | 442 | 560 | 567 | | 574 | 554 |  |
| CCL2 | 7293 | 4329 | 7505 | 7041 | 9386 | 8284 | 12618 | 8469 | 9335 | 18684 | | 19130 | 23427 |  |
| CCL12 | 27372 | 20218 | 26027 | 21447 | 23778 | 26097 | 37669 | 30239 | 35330 | 33372 | | 25953 | 31155 |  |
| CCL22 | 60772 | 75714 | 36656 | 45200 | 43572 | 45669 | 41074 | 41546 | 39817 | 41238 | | 42707 | 46051 |  |
| CXCL9 | 51139 | 46257 | 43433 | 55362 | 57126 | 56173 | 65462 | 53592 | 59018 | 74808 | | 62427 | 60232 |  |
| CCL3 | 714 | 777 | 527 | 392 | 538 | 113 | 0 | 0 | 0 | 447 | | 521 | 684 |  |
| CCL9 | 390735 | 299703 | 344127 | 298379 | 318180 | 307783 | 352001 | 333229 | 354092 | 327154 | | 345746 | 349988 |  |
| CXCL2 | 2706 | 2523 | 4926 | 1923 | 2636 | 1859 | 296 | 1566 | 533 | 18849 | | 17093 | 8999 |  |
| CCL20 | 1361 | 1097 | 1096 | 778 | 196 | 423 | 130 | 381 | 0 | 665 | | 964 | 954 |  |
| CCL19 | 792 | 575 | 572 | 518 | 328 | 318 | 0 | 0 | 0 | 248 | | 363 | 243 |  |
| CXCL4 | 2495 | 2398 | 2603 | 2170 | 1662 | 1755 | 1697 | 1364 | 1982 | 2529 | | 2829 | 2845 |  |
| CCL5 | 82731 | 81224 | 68563 | 52598 | 49790 | 46701 | 72462 | 87464 | 77735 | 57784 | | 46061 | 86909 |  |
| CXCL12 | 1407 | 963 | 1074 | 834 | 760 | 415 | 0 | 0 | 0 | 566 | | 414 | 745 |  |
| CCL17 | 824 | 782 | 621 | 586 | 633 | 629 | 448 | 330 | 625 | 431 | | 503 | 581 |  |
| CCL1 | 10866 | 5332 | 5652 | 8323 | 10707 | 10881 | 8479 | 11371 | 7543 | 10572 | | 12035 | 12054 |  |
| CCL25 | 722 | 509 | 525 | 421 | 309 | 494 | 558 | 318 | 336 | 199 | | 323 | 355 |  |

**Table S2.** Individual pixel density of MAPK Phosphorylation proteins within the tumours at 48 h post treatments (n=3).

| Phosphorylation protein name | untreated | | | BP+aCD47 | | |
| --- | --- | --- | --- | --- | --- | --- |
|  | 1 | 2 | 3 | 1 | 2 | 3 |
| AKT | 2881 | 2976 | 3168 | 3559 | 3034 | 3501 |
| CREB | 957 | 857 | 1116 | 1112 | 989 | 1109 |
| Erk1/2 | 2438 | 2066 | 2780 | 2990 | 2734 | 2995 |
| GSK3a | 2489 | 1926 | 2850 | 2659 | 3177 | 2891 |
| GSK3b | 16719 | 15178 | 17103 | 17877 | 16531 | 16377 |
| HSP27 | 3232 | 2671 | 3686 | 3154 | 3864 | 3614 |
| JNK | 2937 | 2472 | 3308 | 3032 | 2986 | 3316 |
| Mek1 | 1410 | 1204 | 1563 | 1343 | 1745 | 1677 |
| MKK3 | 4093 | 3503 | 4913 | 4376 | 4560 | 4781 |
| MKK6 | 1030 | 1043 | 1068 | 1034 | 1346 | 1271 |
| MSK2 | 1791 | 1458 | 1755 | 2101 | 2277 | 2018 |
| mTor | 4863 | 3785 | 5262 | 5047 | 5667 | 5668 |
| P38 | 4949 | 4526 | 6217 | 5847 | 7267 | 6198 |
| P53 | 2645 | 2369 | 3146 | 3077 | 3552 | 2953 |
| P70S6k | 2041 | 1624 | 2580 | 2029 | 2645 | 2656 |
| RSK1 | 2749 | 2490 | 3065 | 3014 | 3369 | 2824 |
| RSK2 | 1711 | 1325 | 1937 | 1791 | 1925 | 1519 |
